# Fourth-year medical students’ experiences of diagnostic consultations in a simulated primary care setting

**DOI:** 10.5116/ijme.5d5a.77af

**Published:** 2019-08-29

**Authors:** Annamaria Witheridge, Gordon Ferns, Wesley Scott-Smith

**Affiliations:** 1 Cranfield Defence and Security, Cranfield University, Shrivenham, Swindon, SN6 8LA, UK; 2 Division of Medical Education, Brighton & Sussex Medical School, Falmer, Brighton, UK

**Keywords:** Diagnostic reasoning, simulation, assessment, medical students, primary care

## Abstract

**Objectives:**

The aim was to explore the experiences of fourth-year medical students of diagnostic consultations in a simulated primary care setting, in order to gain an insight into the suitability of such simulated consultations for assessing the diagnostic reasoning skills of medical students.

**Methods:**

This single-centre study employed a qualitative, cross-sectional design. Twelve fourth-year medical students volunteered to be filmed across 21 simulated, primary care consultations. The setting closely resembled OSCE stations, with a clinician present at each station monitoring the students’ performance using a station-checklist. Upon completion of each station, participants reflected on their experiences using video-stimulated recall. Interviews were transcribed and analysed using Interpretative Phenomenological Analysis.

**Results:**

The simulated scenarios were often perceived to have limited fidelity with predictable outcomes. At times, preoccupation with the assessment checklist meant that students were more likely to focus on asking questions than interpreting the information they were gaining. Some students felt scrutinized during the consultations, while others struggled to adapt to the time pressure. Overall, the artificial setting seemed to promote a reductionist diagnostic approach and an attitude of ‘ticking boxes’ rather than engaging in active diagnostic reasoning.

**Conclusions:**

The present findings call into question the assumption that observation-based assessment of the performance of medical students during simulated consultations can be reliably used to assess their diagnostic skills. Future studies need to explore how current assessment modalities could be better adapted to facilitate active engagement in diagnostic reasoning.

## Introduction

Diagnostic reasoning refers to the dynamic thinking process of a clinician that underpins the diagnosis and subsequent clinical management of a patient’s condition. Errors in the diagnostic process can be costly and can put the patients’ safety at significant risk.^1^^-^^4^ According to a large-scale, longitudinal study, the majority of diagnostic errors can be linked to faulty reasoning of the clinician, highlighting the importance of sound diagnostic reasoning skills for safe and effective clinical practice.^1^^,^^3^^-^^6^  However, mastering these skills can be challenging for novice clinicians.

A comprehensive report by the General Medical Council found that while newly qualified junior doctors feel prepared for history taking and physical examinations, they do not feel prepared for making a diagnosis in real clinical practice.^7^ Another study reported that junior doctors, early in their postgraduate years, tend to defer diagnostic responsibility, and instead of synthesizing patient information to form a diagnosis, are likely to report the patient’s presenting complaints as the cause of the patient’s problem.^8^ It has been suggested that these challenges around the graduation transition may arise due to the discrepancy between the undergraduate training outcomes and the demands of real practice with respect to diagnostic skills.^9^

In order to ensure that graduates are transitioning into their roles of junior doctors with sufficiently safe diagnostic skills, medical schools need to develop suitable ways to evaluate the diagnostic skills of senior medical students. However, diagnostic reasoning has been a challenging educational target for a number of reasons. First, historically, diagnostic reasoning used to form part of the hidden curriculum in medical education, which means students were expected to develop these skills without being explicitly taught.^10^^-^^14^   Diagnostic reasoning was viewed as a trait that naturally emerges over time, and was not considered an explicit educational target.^15^ Whilst this view has been challenged, there remains a lack of clarity as to how diagnostic reasoning should be incorporated into the undergraduate medical curriculum both in terms of teaching and assessment.^15^^,^^16^

Second, traditional, classroom-based instruction of diagnostic skills may not be sufficient due to the highly context-dependent nature of this skill.^6^^,^^17^^,^^18^ However, the limited patient contact due to tightening ethical regulations in undergraduate medical training may adversely affect the development of diagnostic expertise.^9^ Finally, the appropriate assessment of diagnostic skills is yet to be systematically integrated into the undergraduate medical curriculum.^16^ The skills that are being developed by the students need to be formatively assessed especially as students approach the graduation transition in order to ensure that they can demonstrate sufficient competency in diagnostic skills.

While there has been a scarcity of studies investigating the diagnostic skills of senior medical students,^19^ a recent report by Page and colleagues found that different medical schools incorporate diagnostic reasoning into their curriculum in different ways.^16^ Some medical schools use Objective Structured Clinical Examinations (OSCEs) to assess the diagnostic reasoning skills of their students.Although it has been shown that well-designed OSCEs can reliably assess various clinical skills,^20^ in their current form, they may not be optimal for assessing complex cognitive skills, such as diagnostic reasoning.^16^ Previous studies have indicated that the performance scores on OSCEs do not correlate with diagnostic reasoning ability or diagnostic accuracy,^21^^-^^23^  however, the reasons for this have yet to be explored.

The present study is the first qualitative study to investigate how medical students approach diagnostic consultations in a simulated primary care setting. Primary care consultations may pose an especially high challenge for novice diagnosticians due to the time-pressured nature of the consultations, the undifferentiated, ambiguous symptom presentations, and the limited availability of other diagnostic resources.^19^ The aims of the study were to explore simulated diagnostic scenarios from the perspective of fourth-year medical students, to gain a deeper understanding of the students’ experiences of being faced with a diagnostic task in such setting, and to explore how the OSCE-style simulated setting influences the learners’ experiences.

## Methods

### Study design

This study adopted a qualitative, cross-sectional research design using Interpretative Phenomenological Analysis in order to gain an in-depth understanding of medical students’ experiences of diagnostic reasoning in a simulated primary care setting. Interpretative Phenomenological Analysis focuses on small, homogenous participant samples within a defined context to explore how people make sense of the experiences they have lived through.^24^^,^^25^ This approach was ideally suited for the aims and contextual constraints of the present study. It allowed data collection to be completed prior to analysing the data, which was necessary to allow for multiple interviews on the same day to suit the design of the simulated practice. Methodologies that utilize constant comparison where data collection and analysis are taking place simultaneously, such as Grounded Theory, would not have been feasible here.^26^

#### Setting

The medical school, from which the participants were drawn, has adopted an integrated curriculum with early patient contact during the preclinical years. In the first two years of their training, students attend a GP practice on multiple occasions per year. Students are encouraged to take histories from the patients and perform examinations under direct supervision from their GP tutors. In year 4, general practice training is further enriched through simulated consultations where students can put their skills to practice in a safe, supervised environment with simulated patients playing standardized patient roles. These simulated GP surgeries provided the setting for the present study.

The simulations were set-up and run in a way that closely resembled the OSCEs at the same medical school. There were seven different stations that students were required to visit during the simulation, each set up in separate rooms along the same corridor. At each station, there was an actor present portraying a standardized patient role that is typical of primary care and a facilitating clinician. The standardized patient and the facilitator were both sitting at a table positioned in the middle of the room. Students progressed through the stations in pairs, alternating the leading role at each station while the other student observed. Each simulated scenario lasted for ten minutes, which comprised seven minutes of consultation time and three minutes of feedback. At each station, the facilitators completed an evaluation checklist based on the students’ performance, which was given to the students after the completion of each station as additional feedback.

### Participants and data collection

In order to ensure a homogeneous participant sample for Interpretative Phenomenological Analysis, purposive sampling was used. Medical students from the same cohort at a single UK medical school were invited to participate in the study over the 2015-2016 academic year. Students were sent an email advertisement about the study through an independent administrator, which contained details of the study. There were no exclusion criteria imposed in terms of age, gender, nationality, or otherwise. From the cohort, twelve students volunteered for the study (5 males and 7 females).

The participants were filmed during the simulated consultations. The filming was carried out by placing an iPad on a tripod in the consultation rooms. The software that was used to record the consultation immediately uploaded and stored the videos on a secure website. Once the stations were completed, participants were asked to join the researcher in a quiet room for a semi-structured, reflective interview. During the interview, students reviewed their video footage and reflected upon their experiences of the consultations focusing on their diagnostic reasoning process. The interviews took on average 28 minutes per participant (min: 14 minutes, max: 44 minutes), totalling up to 5 hours and 35 minutes of interview data from 21 simulated consultations.

### Ethical considerations

The project was approved by the Research Governance and Ethics Committee of the medical school where the study took place. Individuals who expressed their interest in the study were provided with detailed information outlining the nature of the study, what was expected of participants, the potential risks and benefits of participating, and how their data would be used. Participants were free to withdraw from the study at any time without having to provide a reason. The only potential risk associated with participating in the study was the possibility of experiencing some anxiety due to being filmed. In order to address this risk, participants were offered an optional post-participation debrief session to discuss any concerns or anxieties that arose during the study. However, none of the participants reported any concerns. Verbal consent was attained from all those appearing on the video footage prior to filming, including the actors, facilitating clinicians and the non-participating student. All data were securely stored and managed in accordance with the university’s data protection and data management policies.

### Data analysis

The interview recordings were transcribed verbatim by the researcher and analysed using Interpretative Phenomenological Analysis based on the guidelines of Smith, Flowers, and Larking.^25^ The analysis began with reading and re-reading the transcripts to increase familiarity with the data. The researcher then moved onto initial, exploratory coding which involves the close, line-by-line investigation of the recorded data. Through this detailed analysis, the researcher assigned descriptive, linguistic and conceptual themes to the data. The next stage of the analysis involved moving away from the transcript itself and focusing on the themes of the exploratory coding in order to identify broad, superordinate themes. This stage of the analysis involved grouping together similar themes (abstraction), elevating initial themes onto superordinate level (subsumption), and dividing initial themes into smaller, but more homogeneous sub-themes (polarization).^25^ These analytic steps were repeated for each transcript. Once the individual analysis of the transcripts was completed, patterns were looked for across the participants to capture the essence of their experiences in a complete and comprehensive way, before presenting it in a rich narrative account that was illustrated with direct quotations from the transcripts.

## Results

The initial analysis of the transcripts revealed three broad, overarching themes. The first concerned the influence of the simulated setting on the diagnostic approach of the medical students; the second related to the diagnostic rules used by the students; and the third one was about the challenges associated with general consultation skills. As the focus of the present paper was the role of context in relation to diagnostic reasoning assessment in undergraduate medical education, only the first theme is discussed in detail here, while the other two themes are described elsewhere.^19^

The following sub-themes emerged relating to the role of context:  the artificiality and predictability of the OSCE scenarios; contextual pressure which encompassed being under scrutiny and being under time pressure; and finally, the lack of reasoning and ticking boxes. These sub-themes are discussed below, illustrated with relevant quotations from the transcripts.

### ‘Artificiality and predictability of scenarios’

The first theme that emerged from the interviews was the students’ perception of the simulated consultations as mock OSCEs. According to most of the students who were interviewed, the setting and the procedures closely resembled those of real OSCEs:

“…it’s very similar to the real OSCE situation” (female, no.5)

The students’ previous experiences with OSCEs meant that they were very familiar with typical OSCE stations and had strong expectations about how certain consultations would unfold:

“…a patient in each of these stations has something that you need to make sure you have gotten out of them” (female, no.4)

“…in an OSCE they usually have like a strong family history” (male, no.8)

This provides them with cues about the “patient” that they would not normally have available during a real consultation. In addition to the familiarity with the general OSCE features, students also seemed to be able to recognize some of the specific stations and identify the specific learning goals they were expected to achieve. Their awareness of these learning goals and expectations seemed to induce an external, third-party focus on the authority figures, whereby students were preoccupied with trying to identify what “they” (i.e. their assessors) wanted them to do:

“…I knew what they wanted of me in this scenario…” (male, no.7)

“…on the mark sheet I am pretty sure they ask you to specifically know what the pills are” (male, no.8)

“…in my mind I was like oh is this the station where you need to like educate them on the problems of antibiotics and you don’t need to give them” (male, no.8)

Their reflection suggests that their attention is directed away from the best interest of the patient towards trying to fulfil the expectations of their assessors. Being fixated on those expectations, students were actively trying to locate physical cues in the consultation room that could be relevant for the diagnostic task. Some students focused on the presence of equipment, props, and documents at each station as possible clues. For example, one student described that in those stations where they were given an examination result as part of the simulated scenario, they were expecting the examination result to be positive and thereby a key to the diagnosis:

“…I was thinking, you know they gave me an ECG, surely there is something I need to find here…” (female, no.3)

Another student mentioned that having specific props around made them feel like they are supposed to use that prop during the consultation:

“…I don’t know why, but you just panic, like right, what equipment I have, I got to use immediately” (female, no.11)

Their accounts suggest that they were interpreting cues that normally would be neutral in real practice, as significant and predictive during the simulation.  This reflects the general perception that the physical set-up of OSCE stations are tailored to the task at hand, and therefore assessing the props of the station may provide cues to their consultations.

Besides the physical cues, another student was talking about consciously trying to identify the specific competency they were expected to demonstrate at each station:

“…in OSCEs you are kind of divided into two stations, you either have a medical patient … or communication patients …, so that’s what I thought was difficult about the beginning of this patient … I wasn’t quite sure if this was a communication station or if it was a medical station…” (male, no.8)

This type of part-task framing of the consultation is different from a real life approach and the students’ focus during the consultation is highly dependent on this framing. Here the student is using their cognitive resources to categorize the stations and focus on demonstrating selected competencies, instead of taking on a whole-task approach as they would in a real consultation.

During the reflections, students commonly referred to the patients as “actors”, and they were contrasting the observed behaviours to how real patients would behave in such scenarios. Pointing out those differences indicated their conscious awareness of the simulated nature of the consultation and suggests low level of immersion in the simulation. Students also had set expectations about how OSCE actors tend to behave during certain consultations and how they tend to answer certain questions.

“…I found that bit really funny, because I think it just showed that he was an OSCE actor, ‘cause he started giving me the pain scores out of ten … So that made it seem very OSCE as opposed to like real life situation.” (female, no.6)

“…Again, I already knew what he was going to say for this question” (male, no.7)

Another aspect of the artificiality of the setting was apparent in the fact that students anticipated the same problem already covered in one station not to recur in subsequent stations:

“…what I am probably thinking is, okay, that’s the ECG one done, what could it be next …” (female, no.4)

This is another instance in which the students rely on a cue that would not be applicable in a real consultation, where multiple patients may present with the same problem in close succession.

Students also had expectations about the general complexity of standard OSCE stations. Therefore, when the station seemed too simple, they kept the inquiry going as they thought there was more to find out.

“…I was thinking like this is quite a simple history, I feel like I have missed something out… It all felt a bit too simple, which isn’t a good sign in an OSCE.” (female, no.6)

Hence their diagnostic enquiry was influenced by the anticipations about the complexity of a standard OSCE station. This approach would obviously not translate to a real-life consultation, as there is no standard complexity for real cases. Finally, OSCE stations were expected to have clear solutions and unambiguous examination findings, unlike real scenarios:

“…I know for an exam, if it was actually supposed to be something blaringly obviously like he is having a heart attack, it probably would be blaringly obvious.” (female, no.6)

The challenge in real primary care consultations is often the ambiguity and vagueness of the information available to the clinicians. One of the students talked about this discrepancy and the anxiety associated with the anticipation of not being able to find an answer to real patients’ problems:

“…with these, there is definitely a clear answer, because they are all, you know, artificially written scenarios, but when you do get into like a proper practice, you might get situations where you do get patients where you do not have any idea what to do. And that’s kind of the scariest thing of it” (male, no.2)

All in all, the artificiality and predictability of the simulated scenarios seemed to promote an unrealistic diagnostic approach, while leaving the students unprepared for dealing with the inherent uncertainty of real primary care consultations.

### ‘Being under scrutiny’

Being observed and assessed added pressure to the consultations. Whilst the simulation did not aim to provide formal assessment, the facilitators were completing evaluation checklists during the consultation based on the students’ performance, which resembled an OSCE-style assessment. The perceived pressure and the students’ desire to appear competent meant that students at times were consciously trying to disguise their weaknesses during the consultations. They thought that giving the appearance of clinical reasoning and covering up their mistakes during the consultations would give the illusion of competence. 

“…I want to do well to show him [the facilitator] that I am capable. So yeah, I feel a little bit pressured, and maybe a little bit distracted as well.” (female, no.5)

“…I was just thinking to keep asking questions to make it look like you know what you are doing. But I guess that is just not clinical reasoning, if I haven’t had anything in my mind to like direct my thoughts.” (female, no.1)

During the simulation, some students experienced high levels of anxiety, which sometimes interfered with their cognitive performance. One student found it difficult to listen to the patient when her anxiety increased, while others mentioned losing their train of thought and not knowing what to do next during the consultation as a result of their anxiety:

“…I go into this almost like a fight-or-flight mode…” (female, no.5)

“…I am nervous, because I really don’t know where I am going and I forgot my, I don’t have my like my train of thoughts, I have completely lost it” (female, no.5)

“…I think I didn’t really hear his questions, because I was still panicking about the ECG, yeah.” (female, no.6)

The perceived pressure of the situation meant that students felt the constant need to do or say something. Some students expressed feelings of helplessness, while others felt like they did not have the chance to stop and think:

“…I feel like I have to keep going doing something, even though I would really like to kind of be like, please would you help me, I am really stuck …” (female, no.11)

“…you can’t even sit there in silence for a minute, you just have to do something…” (male, no.2)

Some students felt that their silence would be interpreted as a sign of incompetence:

“…I don’t want to leave too long of silence, because it makes you seem like you don’t know what you want to ask…” (male, no.8)

Therefore, for some students there seemed to be a perceived pressure to keep the conversation going at all times. However, not being able to pause and think also meant that they were trying to think and talk at the same time, which they found challenging.

### ‘Time pressure’

Another aspect of the perceived pressure was related to the limited time available for primary care consultations. Some students found it difficult to adjust to this type of time pressure:

“…I think the timing is really hard” (female, no.4)

They felt rushed to ask all the general questions that they were taught to cover during standard diagnostic consultations. One of the students expressed their concerns of not having sufficient time to be able to consider differential diagnoses during the consultation, which forced them to stick with their initial diagnosis.

“… I thought it was panic attacks, but I didn’t know about other differentials, other things, and I didn’t think I had enough time to think of those things, so I just ploughed on with my one differential that I had” (male. no.7)

Students found it difficult to apply their knowledge as quickly as they were expected to do during some of the simulated stations. One student explained that given enough time, she would have gone through the diagnostic process in an analytical way, the time pressure did not allow her to do so.

“…if I had lots of time I would be able to almost like write out what I needed to do and really think about that” (female, no.10)

“…you are so time pressured, you can’t really think clearly and…you are only just learning the medical stuff…and then you have got the kind of time pressure to try and remember all anyway, so I would say time is a problem, you just haven’t done it enough to be able to do it quickly” (female, no.10)

One student explained that during their clinical rotations in real clinical practice they have more flexibility with regards to timing. They generally have longer time to think about the clinical problems which they find helpful:

“…in GP surgeries, I have to do actual consultations, there I have a bit more time to think, a bit more leeway” (male, no.7)

Finally, they also realize that in real life practice they will have more flexibility, and they will be able to use their own judgement in determining whether they need to give a patient a few more minutes of consultation time when necessary:

“…it’s difficult, because obviously in GP they only have ten minutes, but really if you need to give a person 15 minutes, in my mind, you give them 15 minutes…” (female, no.4)

Overall, these reflections indicate the difficulties of adjusting to time-constrained diagnostic consultations and also the perceived artificiality of the rigid time limits used in these simulated consultations.

### ‘Ticking boxes instead of diagnostic reasoning’

It was apparent that students perceived certain questions as “OSCE boxes”, which meant they felt obliged to ask them in an OSCE setting, but did not consider them necessary in real scenarios. Students admitted asking these OSCE questions even when they did not find them diagnostically relevant in an attempt to get a higher score on their assessment checklist. However, this discrepancy means that deducing diagnostic ability based on the questions asked during these consultations would be misleading as they do not necessarily reflect the students’ diagnostic reasoning skills.

“…that was more because I thought that would be a mark on the sheet, like for my own diagnosis I didn’t think I need to ask these questions” (female, no.1)

“…in real life I don’t think you would ask the questions about suicide, but in an exam situation you should” (male, no.7)

At times, students did not understand why they were supposed to ask certain questions, as they did not seem diagnostically relevant from their point of view. Knowing the list of questions, but not knowing the diagnostic value of them can give the illusion of diagnostic competence for observers while disguising flaws in the reasoning process or the absence of actual reasoning.

“…I think my diagnostic skills are at a stage today where I know the questions, and I will ask those questions, and sometimes I am not quite sure why I am asking questions exactly….” (male, no.2)

“…always seems to be drummed into us medical students when you’re asking about smoking… I always feel the need to ask, but I never know if it’s a waste of time or not” (male, no.8)

“…I didn’t really know why I was asking that one, it was sort of for completion’s sake…” (male, no.7)

Being preoccupied with ticking boxes while also trying to adjust to the time limit of the consultations, students sometimes rattled through a list of memorized questions without processing the information they received in response to those questions. By asking questions in quick succession, they accumulated information instead of evaluating the diagnostic value of the information, and piecing it all together as they went:

“…I was on a roll, so to speak, I was just thinking I need to ask this, ask this, ask this, so I wasn’t really putting everything he was telling me together at the time. I think because kind of because of the pressure and I am thinking I need to tick some boxes. Like in the yeah I am thinking I need to ask this, I need to ask this, so sometimes I am not necessarily processing the information that I am getting.” (female, no.3)

This indicates that their priority was asking the questions instead of using the information to guide their diagnostic reasoning. Getting through these questions often seemed to be separate from the diagnostic process itself. Ticking the boxes continued even when students thought they have already identified the correct diagnosis and when they saw no real use for further questions:

“…I think I kind of already knew what the problem was, so I was just doing that to tick boxes rather than to actually make it more the diagnosis I think” (female, no.6)

This type of behaviour reflects a pure memory recall exercise instead of opportunity for diagnostic thinking. One of the students explained that it was possible to get full marks on their examinations just by memorizing the station-checklists without actually utilizing much reasoning and critical thinking:

“…sometimes with OSCEs, it is not really testing diagnostic skills, it is testing the fact that you have learned a list of all the different stations that could possibly come up and you have seen all these things, like I am sure I could get full marks on these stations because I would have made one of these [i.e. assessment checklist] myself before I would have checked it. And that is good in a way for learning, but it is not good for like the diagnostic thinking…” (male, no.2)

While it was suggested that students often engage in this type of a ‘box-ticking’ approach during OSCEs, they also realize that this kind of behaviour would not be appropriate in real-life clinical practice:

“…when he said that something had happened in his family I have kind of ignored the point, ticked it off in my mind and then moved on to the next thing. I know it’s not like appropriate, and in front of a patient I would never ever do something like that, it’s just in mind I already kind of like confirmed risk factors for an MI” (male, no.8)

The reflections of another student highlight the discrepancy between the students’ diagnostic approach during OSCEs and consultations with real patients. Her account suggests that the rehearsed nature of questioning patients and the box-ticking behaviour is something she wants to deviate from when interacting with real patients:

“…usually for the OSCEs, because we practice so much, I usually have a set of questions whenever they have a certain kind of disorder, like a mental disorder, the set of questions that you need to ask…. I am trying to remember the questions…At the same time, I want to make it natural, and not just tick boxes, which I tend to do when I am in the OSCEs…” (female, no.5)

Her reflection also suggests that the approach they are trained to use does not transfer to real clinical practice:

“… my first GP visit, when I came in with my kind of tick-box kind of way of asking questions, and you have a real patient … you might almost offend people by like you know, you feel like you are overrunning them with your questions, and so I try to kind of let go of that a little bit.” (female, no.5)

These experiences suggest that the artificiality of these simulated consultations promote a diagnostic approach that is unlikely to be used in real settings, which in turn has implications for the transferability of the gains of this type of simulated practice.

## Discussion

This qualitative study explored fourth-year medical students’ experiences of diagnostic primary care consultations in a simulated setting. The main strength of the study was the use of video-stimulated recall in combination with Interpretative Phenomenological Analysis methodology, which enabled a deep level of qualitative analysis. The findings provide novel insight into how medical students approach diagnostic consultations in a simulated primary care setting that bears a close resemblance to OSCEs.

The main findings here suggest that the contextual pressures of time-restricted, standardized patient consultation, typically used during OSCEs, can have a significant impact on the diagnostic process medical students adopt during these consultations. First, there seems to be a misalignment between the time pressure of these consultations and the students’ diagnostic reasoning skill development. While expert clinicians can often rely on their quick and efficient pattern recognition skills to solve diagnostic problems^27^^,^^28^ medical students do not yet possess elaborate knowledge networks to guide their diagnosis.^29^ Instead they largely rely on analytic reasoning strategies requiring deliberate use of their medical knowledge, which can be time-consuming.^27^ However, the time pressure of the OSCE stations does not always allow for students to rely on those analytical skills. This can mean that when students encounter incongruent information during the consultation that does not support their working diagnosis, they do not have time to re-route their reasoning and therefore end up adopting the initial diagnosis regardless of the incongruence. On the other hand, the duration of GP consultations in real clinical practice often varies.^30^^,^^31^ General practitioners can allow for some extra time when necessary based on the needs of the individual patient.^30^ Therefore, it may be useful to allow students to be assessed on their diagnostic reasoning in a more realistic situation, without such a strict time limit. Having a longer time may encourage more flexible reasoning and critical thinking.

The time pressure together with the checklist-style assessment and the predictability of the simulated scenarios seemed to encourage a reductionist diagnostic approach of rushing through memorized lists of questions, instead of actively engaging in diagnostic reasoning. The recognition of typical OSCE stations can mimics the non-analytical cognitive strategy of pattern recognition,^28^ however, instead of recalling an illness pattern, students are recalling the items on the assessment checklist associated with that specific station, including questions to ask, behaviours to demonstrate and management strategies to use. However, recalling and demonstrating those can take place without necessarily engaging in diagnostic reasoning, which indicates that simulated consultations in a setting like this may not be ideal for diagnostic skill assessment.

When students can achieve points on the checklists by simply asking the right questions, they tend to focus more on what question to ask next, instead of focusing on how to interpret the answers to their questions. This leads to an information accumulating approach instead of letting diagnostic reasoning lead the information gathering. Moreover, in a quest to achieve high marks, students ask questions even when they do not think those questions are diagnostically relevant or necessary, even when they do not know the value of those questions, and even when they would not normally ask those questions in real clinical scenarios. This observed discrepancy between what students overtly verbalize and their underlying cognitions may weaken the validity of observation-based assessment of diagnostic skills. This is in line with previous findings that medical students deliberately change their consultations style during examinations in order to suit the perceived requirements of the assessment.^10^

The students’ motivation to achieve a high score on the evaluation checklist means that their focus is often on trying to figure out what is expected of them in that particular artificial situation, instead of being fully immersed into the simulation and adopting a patient-focused attitude. Their inquiry is often guided by their pre-set anticipations and expectations of typical OSCE stations and patient profiles that they have accumulated over the years. However, this predictability takes away the uncertainty inherent to real clinical consultations. Medical student approaching the graduation transition are often not trained to deal with a realistic level of diagnostic uncertainty that may underlie the diagnostic difficulties they experience as newly qualified junior doctors.^7^ While the present study offers a valuable insight into medical students’ experiences, it also has some inherent limitations. First, the study was carried out in a single medical school, with a small number of participants. While small participant numbers are recommended for studies using Interpretative Phenomenological Analysis, future research in this area is necessary to explore whether the conclusions drawn here are transferable to other settings given the diversity of the undergraduate curriculum and OSCE design among various medical schools. Given the importance of the findings, it would also be important to explore students’ experiences in real assessment settings, in order to explore how to better adapt standardized examinations to facilitate the active use of diagnostic reasoning.

## Conclusions

The constraints imposed by ethical guidelines around the use of real patients in medical training and assessment^32^^,^^33^ and the increasing number of medical students across the UK has led to a greater use of time-constrained, simulated scenarios with checklist-style skill assessment.^9^ However, it has been argued that assessment style in education inherently influences the way students learn,^32^ and therefore the gradual shift away from work-based, apprenticeship-style assessment towards a summative, tick-box style assessment based on simulated practice may be transforming the thinking patterns of medical students, and instead of facilitating flexible, critical thinking, it may foster a limited, reductionist thinking style.

The present findings raise concerns over the use of observation-based, checklist-style assessment of diagnostic reasoning skills in a simulated setting. The reported discrepancy between students’ diagnostic approach in an OSCE-style setting and in real clinical practice questions the predictive power and validity of such assessments. Future research is needed to explore ways to adjust existing examination modalities, so that they are better suited for the assessment of covert, higher-order cognitive skills. Alternatively, new assessment modalities may be required that facilitate active engagement in diagnostic reasoning. There is emerging evidence that senior medical students could benefit from work-based assessment, with consenting patients, as seen in Swedish medical training^35^ or from higher fidelity simulations such as the Safe and Effective Clinical Outcomes (SECO) clinic run by the Department of General Practice and Rural Health at the Dunedin School of Medicine in New Zealand^36^ as possible alternatives to traditional OSCEs.

### Acknowledgments

The research team would like to thank all the individuals who generously shared their time and experiences for the purpose of this research project.

### Conflict of Interest

The authors declare that they have no conflict of interest.
